# Perspectives of clinical microbiologists on antimicrobial stewardship programmes within NHS trusts in England

**DOI:** 10.1186/s13756-015-0090-3

**Published:** 2015-11-16

**Authors:** Chantelle Bailey, Mary Tully, Jonathan Cooke

**Affiliations:** Manchester Pharmacy School, University of Manchester, 1st Floor, Stopford Building, Manchester, M13 9PT UK; Centre for Clinical Epidemiology and Evaluation, Vancouver Coastal Health Research Institute, University of British Columbia, 7th Floor, 828 West 10th Avenue, Research Pavilion, Vancouver, BC V5Z 1 M9 Canada; Centre for Infection Prevention and Management, Division of Medicine, Imperial College, Hammersmith Campus, London, W12 0NN UK

**Keywords:** Antimicrobials, Antimicrobial stewardship, Clinical microbiologists, Semi-structured interviews, Hospitals, NHS Trusts, Content validity

## Abstract

**Background:**

The Antimicrobial Self-Assessment Toolkit for NHS Trusts (ASAT) was developed in England by a National Pharmacist Reference Group of an Advisory Non-Departmental Public Body on Antimicrobial Resistance and Healthcare Associated Infections (ARHAI), in conjunction with the Department of Health. It is intended to identify and evaluate interventions for the promotion and implementation hospital-based antimicrobial stewardship programmes (ASPs). ASAT v16 was produced by iterative validity testing with end-users utilising a sequential exploratory strategy. It was highlighted that there was a need for the inclusion of the domain which targeted the role of clinical microbiologists due to their substantial roles in hospital-based ASP development and implementation.

**Methods:**

This study aimed to investigate the content validity of ASAT v16 and a proposed draft domain for clinical microbiologists and hence produce ASAT v17. From June to September 2011, semi-structured interviews were conducted with ten consultant clinical microbiologists from secondary and tertiary care National Health Service (NHS) Trusts within England. Interviews were conducted until no novel themes were identified i.e., data saturation was achieved. Each interview was digitally recorded and transcribed verbatim and then analysed using a thematic framework that facilitated the identification of emergent themes and sub-themes.

**Results:**

Nine emergent themes were identified which included common enablers and challenges associated with the implementation of effective and sustainable hospital-based ASPs. Key themes included formal governmental mandates, IT infrastructure and also prescribers’ knowledge base of antimicrobial chemotherapy and infectious diseases. Most respondents agreed with the content of ASATv16 and the proposed draft section however they suggested that minor modifications were required to improve question sensitivity and hence reduce measurement error.

**Conclusions:**

Although, the ASAT been through multiple iterations and content validity testing, further modifications were required to produce the next iteration, ASAT v17. Question merging and other minor modifications were conducted as indicated by study findings. This study reinforces the need for stakeholder engagement during the development and implementation of tools that evaluate hospital-based implementation strategies.

## Background

Conceptualisation and development of interventions for the promotion of prudent antimicrobial prescribing has been ongoing for over 20 years [1]. Antimicrobial stewardship can be defined as an ongoing effort by a healthcare institution to optimise antimicrobial utilisation hence improve patient outcomes, ensure cost-effective therapy and reduce adverse sequelae of antimicrobial use (including antimicrobial resistance) [2]. It has been further described as an inter-professional effort, across the continuum of care that involves timely and optimal selection, dose and duration of an antimicrobial, for the best clinical outcome for the treatment or prevention of infection, with minimal toxicity to the patient, and minimal impact on resistance and other ecological adverse events such as *Clostridium difficile (C.difficile)* infection [3].

Hospital-based, organisational interventions used to promote and implement antimicrobial stewardship are collectively known as antimicrobial stewardship programmes (ASPs) [2]. These implementation strategies have been categorised as persuasive, restrictive or structural depending based on the nature of the intervention [4]. The effectiveness of hospital-based ASPs has been extensively investigated and it has been demonstrated that utilising an armamentarium of antimicrobial-related interventions can lead to improved patient outcomes, reduced antimicrobial resistance rates and hence reduce healthcare costs [4]. Also, it has been recommended that collaborative system-wide approaches which incorporate antimicrobial stewardship, infection control and prevention underpinned by patient safety should be undertaken for effective ASPs [5].

Prioritisation of antimicrobial-related interventions is a continual endeavor for national organisations with the remit of producing legislation and policies on healthcare. As such, the Department of Health (DH) and Public Health England have produced Antimicrobial Prescribing and Stewardship Competencies which aims to improve antimicrobial chemotherapy and stewardship within England [6]. This initiative is an essential component of the UK 5-year antimicrobial resistance strategy (2013–2018) [7].

### Overview of the antimicrobial self-assessment toolkit for NHS trusts

In 2008, the first version of the Antimicrobial Self-Assessment Toolkit for National Health Service (NHS) Trusts (ASAT) was developed in England by a National Pharmacist Reference Group of an Advisory Non-Departmental Public Body on Antimicrobial Resistance and Healthcare Associated Infections (ARHAI), in conjunction with the DH [8]. The ASAT has a questionnaire-based format and evaluates composite interventions of hospital-based ASPs including antimicrobial management within the trust, clinical governance, and education and training (see Table 1). In other words, it endeavours to evaluate structure and process measures including policies and procedures utilised for ASP implementation [9–11].

**Table 1:** Headings and descriptions of the domains of ASAT v16 [12]

Domain	ASAT Headings	Description of ASAT Domains
1	Antimicrobial management with the Trust	Examines the Trust Board roles in ensuring good antimicrobial management
2	Operational delivery of antimicrobial strategy	Examines the types of control documents such as antimicrobial guidelines and policies
3	Risk assessment for antimicrobial chemotherapy	Examines patient safety principles that should be undertaken when prescribing antimicrobials
4	Clinical governance assurance	Examines compliance to clinical guidelines and policies by clinical audits
5	Education and Training	Examines the education, training needs and training packages available to antimicrobial prescribers
6	Antimicrobial Pharmacist	Examines the role of the antimicrobial pharmacist to optimise their role in antimicrobial stewardship

### Previous ASAT validity testing

Prior to this study, previous versions of the ASAT were evaluated for face validity and content validity which were used to develop ASAT v16 (see Table 1) [8, 12]. One key finding was that the roles and responsibilities of clinical microbiologists were under-represented and needed to be examined in greater detail in future iterations of the ASAT [12]. Consequently, an additional domain for clinical microbiologists was drafted which contained seven questions that were based on a review of the relevant published literature and guidelines (see Table 2).

**Table 2:** Proposed questions (roles and responsibilities of clinical microbiologists involved in ASPs)

Proposed questions (Clinical microbiologists)	
1	Is there a clinical microbiologist on your hospital’s antimicrobial stewardship committee?
2	Are clinical microbiologists within your hospital involved in the development of antimicrobial policies and guidelines?
3	Are antimicrobial resistance trends used to inform the content of antimicrobial policies and guidelines?
4	Are clinical microbiologists within your hospital in the development of antimicrobial formularies?
5	Are clinical microbiologists involved in ward rounds?
6	Is the reporting of antimicrobial susceptibility testing results in line with formulary choices?
7	Is your hospital actively involved in surveillance or monitoring of antimicrobial resistance trends?

### Clinical microbiologists and ASPs

In the United Kingdom (UK), clinical or medical microbiology is a specialty that encompasses clinical management and the application of scientific laboratory analysis of infection [13]. Hence, the role of consultant medical microbiologists is not entirely laboratory-based, unlike many other countries, including those in Europe and North America. Several guidelines have been published that advocate the role of clinical microbiologists in hospital-based ASPs. For example, these guidelines specifically advocate that the clinical microbiologist should have an integral role in antimicrobial stewardship committees, development of antimicrobial policies and guidelines and ward rounds [9, 14–19].

## Study aims

This qualitative study was conducted as part of an iterative sequential exploratory strategy, to assess the validity of the iterations of ASAT based on the framework proposed by Messick [20]. Hence, this study aimed to investigate the content validity of ASAT v16 and the proposed draft domain for clinical microbiologists and subsequently produce ASAT v17.

## Ethical approval

Ethical approval from the University of Manchester Research Ethics Committee was deemed unnecessary at the time of the study because it was categorised as a service evaluation.

## Methods

### Overview of content validity

Content validity is concerned with item sampling adequacy and examines the extent to which a specific set of items is representative of the content domain [20–22]. In other words, does the ASAT measure what it purports to measure? Can accurate inferences be made about the target population based on ASAT scores?

### Methodological approach

A total of ten semi-structured interviews were conducted with consultant clinical microbiologists who were employed within NHS trusts in England. Sampling based on participants’ specialist knowledge of ASPs was utilised i.e., purposive sampling. Interviews were conducted over a 6-month period i.e., June to September 2011 using a standardised interview protocol [23, 24]. Interviews were conducted until data saturation or no new themes were identified were identified [25–27].

### Qualitative data analysis

Interviews lasted an average of 67 min (range 36–91 min) and were digitally recorded and transcribed verbatim. Each interview was analysed using a thematic framework which facilitated the identification of emergent themes and sub-themes (see Table 3) [24, 28]. Respondents are identified by a number which indicated the order they were interviewed and illustrative quotes are presented so that the reader is able to judge the researcher’s interpretation of the verbal reports.

**Table 3:** Mapping of emergent themes and sub-themes addressed within ASAT v16 and the proposed draft domain

Themes	Sub-themes	ASAT v16	Proposed domain
National prioritisation and initiatives for reducing hospital-acquired infections	Hospital-based initiatives • Promotion and implementation of ASPs	Section 1: Q1.1 to Q1.3	-
	NHS trust board and senior management leadership	Section 1: Q1.1 to Q1.4	-
	Collaboration with other hospital-based initiatives such as infection prevention and control programmes e.g., reduction of HAIs	Section 1: Q1.1 to Q1.3	-
Antimicrobial stewardship committee	Senior management membership with decision-making capacity within NHS trusts	Section 1: Q1.4	Draft question 1
	MDT committee membership including nurses and specialist pharmacists	-	-
	Clinical leadership of antimicrobial stewardship committees	-	Draft question 1
Antimicrobial policies and guidelines	Development procedures • MDT involvement throughout entire process	-	Draft question 2
			Draft question 4
	Update procedures • Incorporation of resistance trends • Effective communication of updates to antimicrobial prescribers	Section 2: Q2.6, Q2.15, Q2.21	Draft question 3
			Draft question 7
		Section 2: Q2.11, Q2.17	
	Accessibility issues	Section 2: Q2.9, Q2.10	-
	Non-standardised regional and international guidelines	-	-
Role of hospital-based pharmacists	Antimicrobial pharmacists	Section 6: Q6.1 to Q6.9	-
	Ward pharmacists	-	-
	Other specialist pharmacists	-	-
	Non-medical prescribers • Increased remit in antimicrobial prescribing	Section 5: Q5.4, Q5.5, Q5.13, Q5.18	-
Current antimicrobial prescribing practices	Empirical prescribing practices • Need for rapid diagnostics		-
	Autonomous prescribing practices by senior clinicians and surgeons	Section 5: Q5.4, Q5.5, Q5.12	-
	E-prescribing • Availability and accessibility of ‘real-time’ data • Antimicrobial consumption data e.g., DDDs • Communicating rationale for prescribing decisions	Section 4: Q4.8 to Q4.9	Draft question 6
Clinical audit programmes	Types of audits e.g., point prevalence, alert audits	Section 4: Q4.1 to Q4.7	-
	Clinical leadership of antimicrobial-related audits	-	-
	Feedback mechanisms to relevant stakeholders e.g., NHS trust boards and antimicrobial prescribers	Section 1: Q1.8	-
		Section 3: Q3.5	
		Section 4: Q4.9 to Q4.12	
	Need for frequent antimicrobial prophylaxis guideline audits	Section 4: Q4.5	-
Prescribers’ knowledge about antimicrobial chemotherapy	Modes of education e.g., formal vs. informal modes	Section 2: Q2.22, Section 5: Q5.1 to Q5.15	Draft question 5
	Knowledge gaps of junior prescribers	Section 5: Q5.1 to Q5.15	-
	Workload implications e.g., out-of-hours requests	Section 2: Q2.23	-
	Deskilling antimicrobial prescribers e.g., lack of ownership of prescribing decisions	Section 5: Q5.1 to Q5.15	-
IT infrastructure	E-prescribing systems	Section 4: Q4.1 to Q4.9	-
	E-auditing systems	Section 4: Q4.1 to Q4.9	-
	Need for improved IT infrastructure within clinical microbiology laboratories e.g., antimicrobial susceptibility testing	Section 2: Q2.11, Q2.17	Draft question 6
Financial resource allocation	Time dedicated to antimicrobial-related duties	Section 6: Q6.3	-
	Specialist staff to bed ratio required for effective antimicrobial stewardship	-	-

*ASPs* Antimicrobial stewardship programmes, *HAIs* Hospital-acquired infections, *MDT* Multidisciplinary team, *NHS* National Health Service, *DDDs* defined daily doses, *IT* information technology

## Results and discussion

Below is a list of themes i.e., enablers and barriers to developing, implementing and sustaining ASPs as identified by clinical microbiologists (see Table 3).

### National prioritisation and initiatives for reducing hospital-acquired infections

Formal mandates for the reduction of hospital-acquired infections including *C.difficile* and methicillin-resistant *Staphylococcus aureus* (MRSA) produced by the DH have been beneficial to ASPs. These mandates have triggered actions for the promotion and implementation of ASPs at the local hospital-level and have involved substantial collaboration with infection prevention and control initiatives. Respondents emphasised that collaborative, hospital-based antimicrobial stewardship frameworks, which were based on effective working relationships and communication between clinical microbiology, pharmacy and infection control and prevention departments and underpinned with robust hospital information technology (IT) infrastructure were needed to meet these targets.

### Antimicrobial stewardship committees

An active antimicrobial stewardship committee which included senior staff members with decision-making capacity was identified as another key enabler. Respondents suggested that senior staff involvement was required to ensure that actions and recommendations generated by the committee were monitored, completed and subsequently reported to relevant stakeholders within NHS Trusts. Furthermore, they recommended that these committees would be enhanced by multidisciplinary membership because each staff group can provide their own unique perspectives on ASPs. Respondents emphasised the need for specialist pharmacists such as transplant pharmacists to be actively involved in the implementation and monitoring of ASPs within their clinical areas.

#### Leadership of antimicrobial stewardship committees

In terms of antimicrobial committee leadership, respondents suggested that committees should be led by medically-trained health professionals only. They indicated that their committees were usually led by non-medical staff who did not have specialist training in infection diagnostics and/or management. Effective ASPs can only be achieved when antimicrobial committees are led by medically-trained staff that understand the role of clinical microbiology laboratories in infection diagnostics.

### Antimicrobial policies and guidelines

#### Development procedures

Active involvement with the development of antimicrobial policies and guidelines was highlighted as a one of the main roles of clinical microbiologists. During the development phase, a collaborative approach that included consultation with senior clinicians and pharmacists from other specialties resulted in increased guideline ‘buy-in’ and compliance from these specialties. A fragmented approach was not recommended because this led to end-users becoming disenfranchised from the guideline development process, which lead to decreased compliance to guideline recommendations. Furthermore, it was recommended that hospitals should utilise external peer-review by practitioners working in other NHS trusts during the development phase of antimicrobial policies and guidelines.

#### Update procedures

Respondents indicated that antimicrobial policies and guidelines that take into account current antimicrobial resistance trends were required for effective antimicrobial stewardship. However, some respondents stated that effective monitoring of current antimicrobial resistance trends can be problematic and may result in delaying guideline updates. Some respondents have adopted horizon scanning methods to ensure that continual monitoring of resistance trends was sustained. However, the identification of the most efficient methods for communicating updates to each antimicrobial prescriber was reported as an ongoing challenge within hospitals. Also, respondents reported that ensuring prescribers used current versions of guidelines was a limitation to prudent prescribing.

#### Accessibility issues

Policy and guideline accessibility were common limitations reported by respondents. Junior doctors were the most prominent antimicrobial prescribers within their hospitals and have stated that guidelines can be quite lengthy and difficult to navigate although available in both paper-based and electronic formats. To address this limitation, some NHS Trusts have developed quick reference guides in multiple formats that contain diagnostic and treatment algorithms for infections including hospital-acquired pneumonias, urinary tract infections (UTIs) and endocarditis.

#### Non-standardised regional and international guidelines

Respondents reported that the absence of standardised regional guidelines and formularies was problematic for sustaining uniformity in prudent antimicrobial prescribing regionally. This was a major issue for junior doctors as they rotated frequently between NHS Trusts as part of the foundation year training. Each NHS Trust has different antimicrobial guidelines and formularies so therefore junior doctors have to adapt and assimilate those organisation’s local practices. Respondents indicated that standardised regional or national guidelines and formularies would help to maintain consistency in antimicrobial prescribing across hospitals.

A number of respondents indicated that they may refer to international guidelines produced by organisations such as Infectious Disease Society of America (IDSA) for diabetic foot, skin and soft tissue infections and some urinary tract infections. However, they have found that some international guidelines have conflicting recommendations for antimicrobial therapy duration and which were based on resistance patterns in the guideline producing countries.

### Role of hospital-based pharmacists

Due to their specialist knowledge, antimicrobial pharmacists were viewed as essential for successful development, implementation and monitoring of ASPs. Respondents suggested that ward pharmacists and specialist pharmacists should have a greater remit in ASPs such as reviewing stop-orders within their clinical areas. However, they recognised that robust mechanisms were needed to support pharmacists when they challenge inappropriate prescribing both junior and senior prescribers. In addition, most respondents recommended that increasing the remit of non-medical prescribers such as podiatrists and nurses would be beneficial to ASPs because they were more adherent to antimicrobial prescribing guidelines.

### Current antimicrobial prescribing practices

#### Empirical prescribing practices

Although, empirical therapy is often initiated pending microbiological confirmation, this prescribing method was reported as an important barrier. Also, respondents suggested that antimicrobial prescribers should incorporate de-escalation of antimicrobial chemotherapy, where indicated. However, they recognised that rapid diagnostics and improved reporting times were required to overcome this barrier and highlighted the need for improved IT infrastructure and related procedures within clinical microbiology laboratories.

#### Autonomous prescribing practices

Respondents reported that autonomous antimicrobial prescribing by senior clinicians and surgeons was still prevalent within their hospitals and expressed concerns about the knowledge base of antimicrobial chemotherapy of these staff groups. Guideline compliance was substandard within these staff groups and challenging these prescribing practices was highly complex. One respondent commented, ‘One of the main negative things is that consultants still see themselves as independent practitioners and if they want to prescribe something for their patient, they do not want to be told otherwise by a microbiologist’ (CM2) Unfortunately, they have observed that these undesired prescribing practices were passed onto junior antimicrobial prescribers and sometimes remained unchallenged.

#### E-prescribing practices

Recognising that suboptimal prescribing is a major challenge to effective antimicrobial stewardship, some respondents have implemented e-prescribing systems within their organisations; to improve the availability and accessibility of ‘real-time’ antimicrobial data. Generally, it was reported that e-prescribing had resulted in improved data quality on actual antimicrobial prescribing practices and therefore NHS Trusts were able to effectively monitor antimicrobial consumption and calculate defined daily doses (DDDs). This method of prescribing has been beneficial to the continuity of care for patients since subsequent prescribers were able to access prescribing information e.g., the rationale for antimicrobial chemotherapy. In-built restrictions have been developed within current e-prescribing systems which are difficult to circumvent by prescribers, for example, ‘clinical indication’ and ‘duration’ were mandatory data fields therefore these fields have to be completed prior to prescribing antimicrobials.

### Clinical audit programmes

Prospective and retrospective antimicrobial-related audits were identified as beneficial approaches for monitoring of the effectiveness of antimicrobial stewardship interventions. These audits were either part of a rolling clinical audit programme which may include point prevalence audits or alert audits. Audits were conducted in response to changes in resistance trends and may focus on specific infections, antimicrobials, wards, prescribers and whether appropriate specimens were sent for diagnostic testing.

Respondents indicated that clinically-led, multidisciplinary or interdepartmental audits resulted in the most significant changes in antimicrobial prescribing practices, especially among junior doctors. Feedback mechanisms utilised by respondents ensured that there was targeted feedback to staff involved with the prescribing pathway and also senior staff with decision making capacity within the trust. As such, antimicrobial audit results and recommendations were fed back to Trust Boards, Drugs and Therapeutics committees, clinical governance committees, medical directors and also relevant clinical teams via quarterly or monthly clinical audit meetings. However, some respondents indicated that their current systems for collecting data on antimicrobial consumption, antimicrobial resistance and sensitivity trends needed to be improved. These limitations were affecting the number of antimicrobial-related audits conducted due to cumbersome data collection processes and hence negatively impacting on the frequency of antimicrobial guideline updates.

Some NHS trusts have implemented locally-developed, evidence-based antimicrobial indicators and checklists for prescribers, antimicrobial quality dashboards and feedback tools for monitoring antimicrobial activity and indicator compliance. In addition, respondents recommended that the frequency of audits on surgical prophylaxis guidelines should be increased since these guidelines were not routinely audited.

### Prescribers’ knowledge about antimicrobial chemotherapy

The provision of education and clinical advice to medical and non-medical prescribers within primary, secondary and tertiary care settings was highlighted as one of their key responsibilities. Educational interventions were conducted on a formal or informal basis in order to increase the sensitisation of prescribers about prudent antimicrobial prescribing. Generally, these interventions were conducted in-person during ward rounds or by telephone however they were unsure which educational mode was the most effective or sustainable.

In addition, respondents expressed concerns regarding the status of undergraduate and postgraduate education about antimicrobial chemotherapy and infectious diseases and suggested that there should be greater emphasis placed on antimicrobial prescribing within the medical curricula. Due to these knowledge gaps, clinical microbiologists indicated that they received a substantial number of requests from antimicrobial prescribers which has significantly impacted on their daily workload. These requests were mainly due to antimicrobial prescribers not taking ownership of their prescribing decisions which led to them becoming increasingly reliant on the advice provided by clinical microbiology, especially during out-of-hours. Respondents cautioned that this process may result in deskilling antimicrobial prescribers. Deskilling was also raised in relation to the utilisation of restrictive interventions because these interventions could possibly inhibit the development of prescribers’ knowledge base about antimicrobial chemotherapy.

### IT infrastructure

Respondents elaborated on the need for improved IT infrastructure within clinical microbiology laboratories, specifically for capturing and reporting antimicrobial susceptibility and resistance data since some hospitals used manual methods to monitor these trends. Some respondents indicated that susceptibility data can be skewed and biased when aggregated. However, respondents emphasised the need for hospitals to produce high quality data because these data are required to track trends, modify guidelines and hence promote prudent antimicrobial prescribing.

### Financial resource allocation

Generally, respondents indicated that their roles within ASPs were transitioning and becoming increasingly collaborative with clinical pharmacy, infection prevention and control teams and other clinical specialties. However, financial constraints such as fixed or reduced hospital operational budgets have resulted in the reduction of time dedicated to antimicrobial-related duties. For example, one respondent commented, ‘time dedicated to the role is a major hindrance… We use microbiology results all the time to alter therapy we have a restrictive policy but now with less time, we can’t look at all the reports coming through …’ (CM5). Most respondents estimated that they dedicated between 1 and 2 days per week for antimicrobial-related duties. In addition, the current ratios of specialist staff to bed ratio were inadequate to cover ASP-related demands, effectively. Respondents suggested that antimicrobial prescribing would significantly improve if they spent a greater amount of time on the wards where they could challenge inappropriate prescribing and act as an educational resource for antimicrobial prescribers.

### Representation of identified themes in ASAT v16 and the proposed domain

This study aimed to investigate the content validity of ASAT v16 and the proposed domain for clinical microbiologists involved in hospital-based ASPs. Table 3, presents an overview of the emergent and subthemes which have been mapped to ASAT v16 and the draft domain. It was observed that ASAT v16 targeted most of the emergent themes raised by respondents. However, there were some themes and sub-themes which were not specifically advocated by the toolkit. For example, although ASAT v16 (Domain 1) included questions targeting whether antimicrobial stewardship committees were part of ASPs, it does not target the leadership of these committees. The evidence base regarding the leadership of these committees was equivocal and therefore was not specifically advocated in the toolkit [4]. ASAT v16 (Domain 2) examined the availability of peer-reviewed, evidence-based guidelines for common infections and surgical prophylaxis and also accessibility, content, review frequency of these documents within NHS Trusts. However, the availability of regional standardised guidelines was viewed as outside the remit of the toolkit. Although, the roles of ward and specialist pharmacists were not specifically advocated by the toolkit, it examined whether these staff groups received education and training on antimicrobial chemotherapy and whether they were competency-assessed as antimicrobial prescribers. The evidence-base was equivocal regarding the leadership of audit with feedback interventions so therefore it was not advocated within the toolkit [4]. However, ASAT v16 (Domain 4) focused on clinical governance and audit structures within NHS trusts including the frequency of guideline audits, monitoring of antimicrobial consumption and audit feedback meetings. Specific systems for diagnostic testing, auditing, education and prescribing were not targeted by the toolkit because it was recognised that ASPs were in different stages of development. Systems such as e-prescribing utilised would be dependent on the availability and allocation of financial and health human resources which could be dedicated to ASPs and hence context-dependent.

The draft proposed domain contained questions which were mapped to emergent themes and it was apparent that these roles were already being conducted by respondents (see Table 3). In addition, respondents agreed that the next iteration i.e., ASAT v17 should contain the section dedicated to clinical microbiologists because their roles were pivotal to the success and sustainability of ASPs. Respondents indicated that clear specifications about their antimicrobial-related roles could assist in the justification for financial and human resources when preparing business cases in the future.

ASAT v16 and the proposed draft domain examine similar measures developed by Antibiotic Strategy International (ABS) Quality Indicators Team for European hospitals [29]. The measures were classified into antimicrobial stewardship services tools, human resources and mandate, health care personnel development, basic diagnostic capabilities, microbiological rapid tests, evaluation of microbiological drug resistance data, antimicrobial consumption control and use monitoring which reinforces that the toolkit have incorporated pertinent components of hospital-based ASPs.

### Implications for ASAT v17

Based on the study findings, modifications were made to ASAT v16 to produce ASAT v17, to improve the validity of the toolkit. Respondents suggested that further minor modifications were required for the proposed domain prior to the incorporation into the toolkit in order to improve question sensitivity and reduce measurement error. Draft questions (Q2 and Q4 (see Table 2) were viewed as measuring the similar concepts because formularies were directly derived from guidelines, hence Q4 was deleted. Also, the question which asked whether clinical advice from clinical microbiologists i.e., Q2.23 was moved from Domain 2 and incorporated into newly created domain. Other modifications included the addition of a glossary of definitions and also signposting ASAT end-users to the publication ‘START SMART then FOCUS’ and similar publications [30].

### Strengths

To the researcher’s knowledge, this is the first study to investigate the perspectives of clinical microbiologists involved in ASPs within NHS Trusts in England. Interviews provided data on the roles of clinical microbiologists and also the enabling and limiting factors that affect the development and sustainability of ASPs within NHS Trusts.

### Limitations

One limitation of this study was due to the small sample (*n* = 10) used to test the content validity of the toolkit. Maximum variability sampling was used so therefore every effort was made by the researcher to obtain a sample of the intended end-users of the toolkit [31]. However, these results could be considered indicative of problems which similar clinical microbiologists may encounter while establishing hospital-based ASPs.

In addition, the respondents for this study were homogeneous and the study did not target other healthcare professionals involved in antimicrobial stewardship. Further research could involve investigating the perspectives of each staff group involved in the antimicrobial prescribing pathway utilising a qualitative approach i.e., those who prescribe, administer and monitor antimicrobials. Other staff groups who may not be directly involved in prescribing decisions could also be targeted such as staff that dispense antimicrobials. These data could provide insight on antimicrobial prescribing from the perspectives of these staff groups and identify the strengths and weaknesses of local ASPs. This study could be conducted in conjunction with an investigation into patients’ perspectives on antimicrobial chemotherapy. Complementary research investigations could focus on the development of antimicrobial prescribing indicators for hospitals [32].

## Conclusion

This was the second study conducted which specifically examined the content validity of the ASAT and concluded that there was sufficient evidence for the inclusion of the domain for clinical microbiologists within ASAT v17. It highlights the need for ongoing stakeholder engagement when developing and implementing ASP-related interventions in hospitals due to the continual need to preserve antimicrobials. With the publication of Critically Important Antimicrobials for Human Medicine, increased prioritisation of ASP-related interventions is becoming significant for sustaining the efficacy of these agents.

## Abbreviations

UK: United Kingdom
NHS: National Health Service
ASAT: Antimicrobial Self-Assessment Toolkit for NHS Trusts
HAIs: Hospital-acquired infections
DH: Department of Health
ARHAI: Antimicrobial resistance and healthcare associated infections sub-group
IT: Information technology
DDDs: Defined daily doses
C.difficile: Clostridium difficile
MRSA: Methicillin-resistant *Staphylococcus aureus*
ASP: Antimicrobial stewardship programme
CM: Clinical microbiologist
